# Using household economic survey data to assess food expenditure patterns and trends in a high-income country with notable health inequities

**DOI:** 10.1038/s41598-022-26301-z

**Published:** 2022-12-15

**Authors:** Nhung Nghiem, Andrea Teng, Christine Cleghorn, Christina McKerchar, Nick Wilson

**Affiliations:** 1 Department of Public Health, University of Otago, Wellington, New Zealand; 2 Department of Population Health, University of Otago, Christchurch, New Zealand

**Keywords:** Diseases, Health care

## Abstract

This study aimed to identify dietary trends in Aotearoa New Zealand (NZ) and whether inequities in dietary patterns are changing. We extracted data from the Household Economic Survey (HES), which was designed to provide information on impacts of policy-making in NZ, and performed descriptive analyses on food expenditures. Overall, total household food expenditure per capita increased by 0.38% annually over this period. Low-income households spent around three quarters of what high-income households spent on food per capita. High-income households experienced a greater increase in expenditure on nuts and seeds and a greater reduction in expenditure on processed meat. There was increased expenditure over time on fruit and vegetables nuts and seeds, and healthy foods in Māori (Indigenous) households with little variations in non-Māori households. But there was little change in processed meat expenditure for Māori households and expenditure on less healthy foods also increased over time. Routinely collected HES data were useful and cost-effective for understanding trends in food expenditure patterns to inform public health interventions, in the absence of nutrition survey data. Potentially positive expenditure trends for Māori were identified, however, food expenditure inequities in processed meat and less healthy foods by ethnicity and income continue to be substantial.

## Introduction

Dietary risk factors are one of the most important risk factors for non-communicable diseases (NCDs) worldwide, accountable for 11 million premature deaths and the loss of 225 million disability-adjusted life years (DALYs) in 2017^1^. These risk factors contribute to cardiovascular disease (CVD), diabetes, and cancer; which are among the top leading causes of deaths globally^2^. Household food expenditure surveys are increasingly used to monitor changes in dietary patterns internationally as individual nutrition survey data are often lacking and such nutrition surveys are expensive^3–7^. Employing food expenditure data, studies have suggested that urbanisation, industrialisation and globalisation among other factors have shifted dietary patterns towards more processed foods^8–12^. Food-insecurity is also highly correlated with total household food expenditure^13,14^, and low-income households often lack access to nutritious foods^15^.

In Aotearoa New Zealand (NZ), the NZ Burden of Disease Study shows that nutrition and obesity factors contribute to 18.6% of total health loss (in DALYs). In addition, diet and obesity related diseases are unequally distributed by ethnicity and deprivation, with Māori, Pasifika and groups with low socio-economic position at a higher risk of having obesity^16^ and NCDs^17^. Much of this health loss and premature death could be prevented by improved diet and addressing the obesogenic environment that encourages unhealthy nutrition (among other interventions such as increasing physical activity). Dietary patterns that are high in sodium, low in fruits and vegetables, low in nuts and seeds, high in processed meat and high in sugar-sweetened beverages are the major risk factors for NCDs including CVD, diabetes and cancer^1,18^.

Despite the relative importance of dietary risk factors in generating health outcomes, the most recent Adult Nutrition Survey in NZ was over a decade ago (2008/09) and for children it was around two decades ago (2002)^19^. For this reason, there are no recent representative data on trends in dietary patterns in NZ (but note that the NZ Health Survey included food frequency questions in 2019/20), nor changes in the distribution of diet by social factors.

Furthermore, different data sources have different strengths and weaknesses. Trends in household food expenditure do not directly indicate individual diet, but are particularly relevant to concerns about food security and the cost of healthy food, particularly for low-income households (eg, the proportion of income spent on food). Expenditure data can also be used to examine changes in types of food bought over time.

The linkage of the NZ Household Economic Survey (HES) data (2006/07, 2009/10, 2012/13)^20^ creates repeated cohorts of nationally representative data. The HES contains detailed information about household food expenditure, alcohol expenditure, tobacco expenditure, and other non-food household expenditure; and is implemented every 3 years^21^. HES data can also contribute information on food consumption patterns^4,22^. More importantly, the household economic information has been routinely collected; it is extremely cost-effective if these data can also be used to analyse changes in household diets to assist public health interventions. We therefore aimed to explore the trends and social patterns in NZ household dietary expenditure using national representative linked HES data in NZ. In particular, the purpose of the study was to analyse dietary expenditures between Māori (Indigenous population) and non-Māori, and low-income and high-income population groups.

## Methods

We used data from three HES waves (2006/07, 2009/10, 2012/13) with a total of 9030 households^20,23^. These samples were randomly drawn from the total NZ resident population. In this survey, a ‘household’ is a group of people who share a private dwelling and normally spend four or more nights a week in the household. Household members must share consumption of food or contribute some portion of income towards the costs for living as a group. Individuals included in the sample are all usually resident individuals living in private dwellings in urban and rural areas in NZ. Data are collected by survey interviewers who visit participated households and complete face-to-face interviews with each eligible household member. The HES survey has three related-components for this study: a household demographic questionnaire, a housing expenditure questionnaire with a 2-week expenditure diary, an income questionnaire for each household member aged 15 and above. Household expenditure includes: food (~ 500 food items), alcohol, tobacco, transportation, housing, health, education, recreation and culture, and other goods and services. The sample for the HES was selected using a two-stage stratified cluster design, which households are sampled on a statistically representative random basis, from rural and urban areas throughout the country. Further information about this survey methodology and data are described elsewhere^24^. There is a strong correlation between what individuals report about individual income in the HES and their actual income recorded in the Inland Revenue data^23^.

Three specific food groups were examined: fruit and vegetables (including frozen), nuts and seeds and processed meat, as per Global Burden of Disease (GBD) Study 2017 data^1^, Ni Mhurchu et al.^25^ which used HES data, and our previous work^26^. These food groups were selected because they are major risk factors for CVD^1^ and can be categorised in the HES. An overall healthy food group was defined using the nutrient-profiling criteria as per Waterlander et al.^27,28^ and included all fresh fruit and vegetables (plus frozen fruit and vegetables), fresh seafood, nuts and seeds, whole grains, milk, legumes and bottled water. A less healthy group was defined as all the remaining food and beverages, such as sugar-sweetened beverages, snack food such as potato chips, confectionary, and takeaway foods. Households with zero total food expenditure or negative total income were excluded from the analysis.

The main outcomes of interest for each food group were expenditure per person per year (2013 NZ$, annual values were provided by the data provider Stats NZ), expenditure as a proportion of total food expenditure, and expenditure relative to total income, all obtained from the HES data. We deflated income and expenditure to get comparable measurements across HES waves by converting all monetary values to the NZ$2013 values (e.g., multiplying expenditure in 2006 with annual inflation rates from 2006 to 2012)^29^. We calculated mean expenditure and its standard error (se) for total food and each food sub-group in each HES wave. Expenditure by average household income-level per person (high- and low-income were defined as above and below the median for the HES survey respectively) and by household ethnicity (whether any household members self-identified as Māori or not) were also calculated. Expenditure trends, relative risks and significance levels were estimated using linear regressions, employing survey weights and adjusting for sampling structure. Independent variables for these linear regression models were survey year and either household income-level per person or household ethnicity.

Data were extracted using SQL version v.18.8, and were further processed and analysed in R using the ‘survey’ package, version R.3.6.0.

### Data protection

Anonymised individual data were obtained from Statistics NZ under the security and confidentiality provisions of the Statistics Act 1975, https://www.stats.govt.nz/integrated-data/integrated-data-infrastructure/, and the methods used were approved by the Ethics Committee reference number HD19/057 by University of Otago, New Zealand. The need for informed consent was waived by the University of Otago Human Ethics Committee, New Zealand due to retrospective nature of the study.

### Ethical approval

Ethics Committee reference number HD19/057 by University of Otago, New Zealand. All methods were carried out in accordance with relevant guidelines and regulations. All experimental protocols were approved by University of Otago, New Zealand.

## Results

There was a total of 9030 household survey participants evenly distributed across three HESs (2901 in 2006/07, 3126 in 2009/10, 3003 in 2012/13). Data on food expenditure was not available for 0.76% of households and they were excluded from the analysis; and 0.63% households had no income and so were unable to be included for the expenditure as a proportion of income outcome measure. Mean household income of the total sample was NZ$37,700.

Māori households accounted for around 17% of the total sample (1520 households). Māori households across all cohorts had larger differences relative to non-Māori households in income, age, household size and the number of households with children. Māori households had a lower mean income (NZ$30,400 per capita) compared to non-Māori (NZ$39,200 per capita). Māori households spent less on food per capita NZ$3490 (11.5% of total income) compared to NZ$4230 (10.8% of total income) for non-Māori. The Māori population appeared to be younger than non-Māori population with a mean age of 28.8 versus 39.5 years. Māori households tended to have more people, with a medium size of 3.09 compared to 2.43 for non-Māori and had a greater percentage of households with children; 48.8% compared to 34.5% non-Māori households with children (see Appendix A). For further characteristics of the survey samples see Table 1.

**Table 1:** Characteristics of repeated New Zealand Household Economic Survey samples.

Specific metrics	Total sample^a^	2006/07	2009/10	2012/13
**General characteristics**				
Households sample size (n)	9030	2900	3130	3000
Households included in this study (n)	8910	2850	3090	2960
Households excluded because they had no food expenditure data (n, %)	69 (0.76%)	24 (0.83%)	12 (0.38%)	30 (1%)
Households excluded because they had no income data (zero or negative income)	57 (0.63%)	24 (0.83%)	21 (0.67%)	9 (0.3%)
Households members (n)	22,700	7340	8000	7310
Mean age (years)	37.3	36.1	37.1	38.9
**Household composition**				
Households with children (n)	3340 (37.5%)	1080 (37.7%)	1200 (38.8%)	1070 (36%)
Households with members aged 65 or more years old (n)	1760 (19.7%)	507 (17.8%)	579 (18.7%)	672 (22.7%)
Mean household size	2.54	2.57	2.59	2.47
**Sex**				
Male (n)	10,900	3540	3850	3510
Female (n)	11,800	3800	4160	3810
**Household income (NZ$ in 2013 value/year) per capita**				
Mean	37,700	37,500	37,000	38,500
Median	28,400	28,200	28,400	28,600
Low-income				
Mean	24,900	24,500	24,800	25,400
Median	20,900	20,800	20,800	21,100
High-income				
Mean	57,900	57,100	55,940	60,800
Median	47,300	47,100	45,900	49,100
Māori income				
Mean	30,400	32,500	27,500	31,500
Median	23,200	23,400	22,800	23,300
Non-Māori income				
Mean	39,200	38,500	39,100	39,900
Median	29,700	29,300	30,100	29,500
**Household ethnicity**				
Households with any Māori member (n)	1520	471	552	492
Households with no Māori members (n)	7390	2380	2540	2470

^a^Numbers may not add up exactly as they were randomly rounded to meet confidentiality requirements. All values in this Table were unweighted.

### Expenditure trends

Annual household food expenditure per capita (in 2013 NZ$) by food group for three HES waves: 2006/07, 2009/10, and 2013/13 are described in Table 2.

**Table 2:** Annual expenditure per capita (in 2013 NZ$) by food group, income-level and ethnicity in three HES waves: 2006/07, 2009/10, and 2013/13^a^.

Food group	Population group	2006/07 (2013 NZ$, se)	2009/10 (2013 NZ$, se)	2012/13 (2013 NZ$, se)	Relative change in food expenditure every three years (%: mean (se))
All food	Total sample	3990 (64.6)	4130 (49.1)	4080 (60.9)	1.13 (1.04)^b^
	Low-income (less than median)	3370 (62.5)	3740 (67.5)	3580 (64.2)	3.11 (1.33)
	High-income (more than median)	4840 (1030)	4720 (84.2)	4840 (97.7)	0.02 (1.47)
	Māori	3240 (116)	3420 (131)	3750 (129)	7.82 (2.69)
	Non-Māori	4170 (69.7)	4310 (55)	4160 (68.7)	− 0.12 (1.17)
Fruit and vegetables	Total sample	437 (9.61)	406 (8.10)	425 (9.21)	− 1.28 (1.50)^b^
	Low-income	399 (11.3)	378 (10.9)	393 (10.6)	− 0.75 (1.95)
	High-income	490 (14.8)	449 (11.8)	474 (12.7)	− 1.63 (2.00)
	Māori	273 (14.9)	275 (13.3)	324 (16.9)	9.34 (4.14)
	Non-Māori	477 (10.8)	440 (9.25)	450 (10.4)	− 2.83 (1.57)
Nuts and seeds	Total sample	31 (1.79)	36 (2.01)	34 (1.69)	5.85 (3.57)^b^
	Low-income	27 (2.37)	31 (2.77)	29 (2.09)	3.70 (5.85)
	High-income	36 (2.49)	43 (3.09)	42 (2.68)	8.33 (5.08)
	Māori	14 (2.02)	18 (2.92)	21 (2.78)	25.0 (12.30)
	Non-Māori	35 (2.07)	40 (2.34)	38 (1.96)	4.29 (4.07)
Processed meat	Total sample	204 (4.63)	199 (4.70)	196 (4.81)	− 2.01 (1.62)^b^
	Low-income	189 (6.07)	191 (6.76)	189 (5.78)	0.00 (2.22)
	High-income	226 (7.24)	211 (6.52)	207 (7.92)	− 4.20 (2.37)
	Māori	191 (9.98)	186 (10.9)	187 (10.1)	− 1.05 (3.72)
	Non-Māori	208 (5.34)	202 (5.36)	198 (5.57)	− 2.40 (1.85)
Healthy foods	Total sample	741 (14.6)	716 (12.2)	715 (13.1)	− 1.71 (1.29)^b^
	Low-income	689 (16.7)	678 (15.7)	677 (15.7)	− 0.87 (1.67)
	High-income	814 (22.1)	774 (19.2)	774 (18.9)	− 2.46 (1.79)
	Māori	507 (25.3)	500 (20.3)	551 (28.6)	4.34 (3.77)
	Non-Māori	798 (16.1)	770 (13.9)	755 (14.3)	− 2.69 (1.35)
Remaining less healthy foods	Total sample	3250 (56.4)	3410 (43.8)	3360 (54.1)	1.79 (1.11)^b^
	Low-income	2680 (52.8)	3060 (60.5)	2910 (58.0)	4.14 (1.46)
	High-income	4020 (91.4)	3950 (74.7)	4070 (87.4)	0.52 (1.57)
	Māori	2730 (101)	2920 (121)	3200 (122)	8.48 (2.91)
	Non-Māori	3370 (60.9)	3540 (48.8)	3400 (60.5)	0.49 (1.27)

^a^Numbers may not add up exactly as they were randomly rounded to meet confidentiality requirements. ^b^Values in this column were derived using linear regressions with the survey year as the only independent variable, but no changes were statistically significant. All values in this table were calculated or estimated using survey weights.

#### Overall trends

Total household food expenditure was increasing slightly from an average of NZ$3990/person (se: 64.7) in 2006/07 to NZ$4080 (se: 60.9) in 2012/13, after adjusting for inflation. Relatively, total food expenditure appeared to increase by 1.13% over 3 years or 0.38% per year between 2006 and 2012. Expenditure on fruit and vegetables (− 1.28% change), processed meat (− 2.01%), and healthy food (− 1.71%), appeared to decrease slightly over time. Expenditure on less healthy foods appeared to increase by 1.79%, and nuts and seeds by 5.85%. These estimated trends in expenditure had wide uncertainty (se > 50% of the mean) and were not statistically significant (based on p-values).

#### Trends by income

Total annual food expenditure for low-income households appeared to increase by 3.11% to NZ$3580 (se: 64.2) in 2012/13, whereas that figure for high-income households remained stable at NZ$4840 (se: 97.7). However, there was a peak in low-income household spending in 2009/10, and at the same time a dip in high-income household spending, which are masked when considering linear trend results. Both types of households appeared to slightly reduce their expenditure on fruit and vegetables, and on healthy foods. Expenditure on nuts and seeds appeared to increase more in high-income (8.33%, se: 5.08) than low-income households (3.70%, se: 5.85). There was a reduction in high-income household expenditure on processed meat over the years by − 4.20% (se: 2.37) not seen in low-income households (0.00% change, se: 2.22).

#### Trends by ethnic group

Total food expenditure for Māori households increased by 7.82% to NZ$3750 (se: 129.8) in 2012/13, but that for non-Māori households slightly reduced (albeit not statistically significant). Māori households increased their expenditure on fruit and vegetables (9.34%, se: 4.14), nuts and seeds (25.0%, se: 12.3), but there was little change in expenditure on processed meat (− 1.05%, se: 3.72). Māori households appeared to increase spending on healthy foods (4.34%, se: 3.77%), whereas non-Māori households decreased their spending on healthy foods (− 2.69%, se: 1.35%). Further details of these food expenditures are provided in Table 2.

Table 3 compares food expenditure by income-level and household ethnicity (relative risks) in each wave. Low-income and Māori households spent less money on all food categories in all years compared to high-income and non-Māori households respectively, and almost all of these differences were statistically significant.

**Table 3:** Relative risks^a^ (%) in household food purchase expenditure for low-income as proportion of high-income, and Māori as proportion of non-Māori households, Household Economic Surveys 2006/07, 2009/10, and 2012/13.

Food group	Population group (as proportion of comparator)	Relative risks in 2006/07 (%: mean (se))	Relative risks in 2009/10 (%: mean (se))	Relative risks in 2012/13 (%: mean (se))
All food	Low-income (high-income)	70 (3)***	79 (2.76)***	74 (2.84)***
	Māori (non-Māori)	78 (3.4)***	79 (3.69)***	90 (3.69)**
Fruit and vegetables	Low-income (high-income)	81 (4.19)***	84 (3.93)***	83 (3.59)***
	Māori (non-Māori)	57 (4.72)***	63 (4.38)***	72 (4.97)***
Nuts and seeds	Low-income (high-income)	76 (11.08)***	73 (12.08)***	68 (9.98)***
	Māori (non-Māori)	40 (11.34)***	45 (12.29)***	55 (11.23)***
Processed meat	Low-income (high-income)	84 (4.74)***	91 (4.8)**	91 (4.97)*
	Māori (non-Māori)	92 (5.73)	92 (6.34)	94 (6.07)
Healthy foods	Low-income (high-income)	85 (3.52)***	88 (3.48)***	87 (3.29)***
	Māori (non-Māori)	64 (4.34)***	65 (3.77)***	73 (4.7)***
Remaining less healthy foods	Low-income (high-income)	67 (3.26)***	78 (2.98)***	71 (3.1)***
	Māori (non-Māori)	81 (3.61)***	83 (4.08)***	94 (4.1)

^a^Values in this table were derived using linear regressions with the survey year and income-level/ethnicity as independent variables. *,**,***Denote statistical significance at the 10%, 5% and 1% levels, respectively.

The gap in expenditure between low- and high-income households (where high-income households spend more) increased over time for nuts and seeds and decreased over time for processed meat. Changes were less clear for other food groups.

The gap in expenditure between Māori and non-Māori households appeared to decline over time for fruit and vegetables, nuts and seeds, and healthy food. The gaps remaining, however, were wide and significant (with Māori households spending 72%, 55%, and 73% respectively of what non-Māori households spent in 2012/13). Similarly, Māori households in 2006 reported a lower expenditure on less healthy foods (81%) than non-Māori, however expenditure increased over time to become 94% (CI 86–102) of the level of non-Māori households in 2012–13.

### Proportions of household food group expenditure out of total food expenditure

Table 4 presents proportion of specific food *group* expenditure out of *total* household food expenditure by income-level and ethnicity for three HES waves. Overall, expenditure on fruit and vegetables accounted for 11% of the total food expenditure, less than 1% for nuts and seeds, 5% for processed meat, with 19% towards healthy foods and 81% for less healthy foods. There were small fluctuations in the proportions of specific food group expenditure out of total food expenditure, however, except for expenditure on nuts and seeds by Māori households (increased from 0.34% in 2006/07 to 0.49% in 2012/13), these changes were not statistically significant.

**Table 4:** Means of proportion of specific food expenditure out of total annual household food expenditure by food group, income-level and ethnicity in three HES waves: 2006/07, 2009/10, and 2013/13.

Food group	Population group	Proportion of food specific expenditure out of *total food expenditure* (%; mean(se))		
		2006/07	2009/10	2012/13
Fruit and vegetables	Total sample	11.1 (0.20)	10.1 (0.17)	10.8 (0.21)
	Low-income	11.8 (0.28)	10.3 (0.23)	11.2 (0.30)
	High-income	10.2 (0.25)	9.8 (0.23)	10.2 (0.23)
	Māori	8.1 (0.41)	8.4 (0.32)	8.9 (0.43)
	Non-Māori	11.9 (0.23)	10.5 (0.19)	11.3 (0.23)
Nuts and seeds	Total sample	0.63 (0.03)	0.77 (0.04)	0.79 (0.04)
	Low-income	0.61 (0.05)	0.74 (0.05)	0.75 (0.06)
	High-income	0.67 (0.04)	0.81 (0.05)	0.86 (0.05)
	Māori	0.34 (0.05)***	0.46 (0.06)***	0.49 (0.06)***
	Non-Māori	0.71 (0.04)	0.85 (0.04)	0.87 (0.04)
Processed meat	Total sample	5.45 (0.13)	5.21 (0.11)	5.14 (0.12)
	Low-income	5.77 (0.20)	5.54 (0.16)	5.62 (0.17)
	High-income	5.01 (0.15)	4.73 (0.13)	4.42 (0.15)
	Māori	6.73 (0.40)	5.88 (0.28)	5.42 (0.29)
	Non-Māori	5.14 (0.12)	5.04 (0.12)	5.08 (0.13)
Healthy foods	Total sample	19.5 (0.29)	18.3 (0.25)	18.8 (0.27)
	Low-income	21.2 (0.41)	19.3 (0.33)	20.0 (0.38)
	High-income	17.2 (0.35)	16.9 (0.35)	16.9 (0.32)
	Māori	16.0 (0.55)	16.1 (0.48)	16.0 (0.66)
	Non-Māori	20.4 (0.34)	18.9 (0.28)	19.5 (0.29)
Remaining less healthy foods	Total sample	80.4 (0.29)	81.6 (0.25)	81.1 (0.27)
	Low-income	78.7 (0.41)	80.7 (0.33)	79.9 (0.38)
	High-income	82.7 (0.35)	83.0 (0.35)	83.0 (0.32)
	Māori	83.9 (0.55)	83.8 (0.48)	83.9 (0.66)
	Non-Māori	79.6 (0.34)	81.0 (0.28)	80.5 (0.29)

*,**,***Denote statistical significance at the 10%, 5% and 1% levels, respectively.

Table 5 compares differences in the above expenditure proportions by income-level and ethnicity for each time-period. Low-income households spent greater proportions of their food budget on fruit and vegetables, processed meat and healthy foods than high-income households (peaking at 27% more than high-income households). Māori households spent greater proportions of the food budget on less healthy food especially in the first time period (30% more on processed meat and around 5% more on less healthy food in 2006/07) compared to non-Māori households; and a lower proportion of the food budget on healthy food (68% fruit and vegetables, 49% nuts and seeds, and 80% for healthy foods in 2006/07 of the level in non-Māori households). These patterns largely persisted over the years, but with some nutrition-favourable trends for Māori households (e.g., increased proportion on fruit and vegetables and decreased proportion on processed meat).

**Table 5:** Relative risks (%) in proportion of household food purchase expenditure for low-income as proportion of high-income and Māori as proportion of non-Māori households, HES 2006/07, 2009/10, and 2012/13.

Food group	Population group (as a proportion of comparator)	Relative risks in proportion of food *group* expenditure out of *Total food expenditure (%: mean (se))*		
		2006/07	2009/10	2012/13
Fruit and vegetables	Low-income (high-income)	116 (3.33)***	106 (3.18)*	109 (3.19)***
	Māori (non-Māori)	68 (4.76)***	80 (3.8)***	78 (4.57)***
Nuts and seeds	Low-income (high-income)	91 (9.97)	91 (9.83)	89 (9.54)
	Māori (non-Māori)	49 (11.26)***	55 (11.34)***	56 (10.52)***
Processed meat	Low-income (high-income)	114 (4.51)***	117 (3.72)***	127 (3.89)***
	Māori (non-Māori)	130 (7.5)***	117 (5.6)***	106 (5.84)
Healthy foods	Low-income (high-income)	124 (2.6)***	113 (2.42)***	118 (2.42)***
	Māori (non-Māori)	80 (3.73)***	85 (3.09)***	82 (3.89)***
Remaining less healthy foods	Low-income (high-income)	95 (0.66)***	97 (0.56)***	96 (0.58)***
	Māori (non-Māori)	105 (0.85)***	103 (0.66)***	104 (0.86)***

^a^Values in this table were derived using linear regressions with the survey year and income-level/ethnicity as independent variables. *, **, ***Denote statistical significance at the 10%, 5% and 1% levels, respectively.

## Discussion

This analysis identified some nutritionally-favourable expenditure trends for Māori. There was increased expenditure on fruit and vegetables, nuts and seeds, and on the healthy food category in Māori households, although expenditure also increased on less healthy foods as well. As a result, the relative gap in health-related food expenditure between Māori and non-Māori households declined over time. However, a stark difference in expenditure remained, with around half to a quarter lower expenditure by Māori households in the healthy food groups. The trends in fruit and vegetables and nuts and seeds were similar for expenditure as a proportion of the food budget and as a proportion of income; although there was no significant change in these indicators for Māori expenditure on healthy foods. Some findings were less favourable from a nutritional perspective. There was little change in processed meat expenditure for Māori households and it remained at a level just slightly less than non-Māori households. Less healthy food expenditure as a proportion of total food expenditure changed little over time, but as a proportion of income it increased in Māori households more than non-Māori households (see Appendix B). Māori households differed significantly in income, household size and the percentage of households with children, so these factors may mediate the association between ethnicity and food expenditure.

Income inequities in food expenditure appeared to be relatively stable over time, however there were some potentially concerning trends from a nutrition perspective (unadjusted for ethnicity). High-income households experienced a greater increase in nuts and seeds expenditure (increasing the gap). Low-income households had a greater increase in less healthy food expenditure, but it remained almost a third less than high-income households. Higher-income households also had a greater reduction in expenditure on processed meat (reducing the gap because expenditure remained higher than for low-income households).

Approximately 60% of the food available in NZ supermarkets have been classified as ‘ultra-processed’^30^. In our study, we found roughly 81% of total food expenditure was spent on less healthy foods (Table 4). A diet that meets the NZ food and nutrition guidelines was estimated to cost approximately an extra $13.50 per week for a family of four^31^. It has been estimated for families on income support in NZ or on low wages, a healthy diet is unaffordable as it may cost up to a half of total household expenditure^31^. However, our study found that on average, low-income households spent approximately 17% of their total income on food, including 3% on healthy foods and the rest on less healthy foods (Appendix B, Table B1). NZ’s grocery prices are also relatively high overall. In 2017 NZ ranked as the sixth highest priced grocery market in the OECD^32^, while its income per capita is below the OECD average^33^. New Zealanders also appear to spend a relatively large proportion of their income on food and groceries in comparison to other high-income countries^32^. The country’s ‘supermarket duopoly’ may be a contributing factor and this currently being investigated by the relevant NZ Government agency: the Commerce Commission (with preliminary findings suggested that the food market competition was not effective for consumers).

Our study has the following contributions to the literature: (1) providing further evidence that HES survey data can be used for understanding trends in food expenditure patterns that will help to assist policy interventions to reduce diet-related disease burden inequities; (2) showing the level of improvement in diet inequities by ethnicity and income-level in NZ; and (3) recommending policy implications for improving nutrition and reduce inequities in diet-related diseases in a high-income country context.

### Strengths and limitations

This study was the first (that we know of) to use HES data for informing food expenditure patterns in the NZ setting. Strengths of the HES data are that it is repeated every 3 years, is representative of the whole NZ population, and is a validated tool for informing economic policy. It appears to be extremely cost-effective to use these data to analyse changes in household diets in order to inform public health interventions. However, it should be noted that even though the Māori sample is at least large enough to perform the analysis, it does not provide equal explanatory power for Māori compared to non-Māori. These data included 2-week diaries for household expenditure so it reasonably covers the major food groups. However, there were some minor food category changes over the years in HES. There were also no data on non-commercial sources of food e.g., home-grown, food from the wild (gathering kai moana [e.g., shellfish], fishing, and hunting), gifting of food (a feature of both Māori and Pasifika cultures), and provision of free food via school breakfast and lunch programmes (for low-income schools). Most importantly, there was no food purchase quantity data collected in this dataset and no accounting for food wastage (which can often be large in the fruit and vegetable category^34^). Finally, our study did not adjust for regional differences in income as the main focus of the paper was to investigate the relative changes in food expenditure. As a result, low-income households in our analysis might include more people living in remote or economically disadvantaged regions. In this sense, our study results were conservative.

### Research and policy implications

From a health and nutritional perspective there is a need to keep the HES and have a routine plan to analyse the food expenditure aspects. But it is also desirable to further contextualise the HES data with other routine data collection (e.g., supermarket sales data—albeit somewhat expensive to purchase from commercial providers). Also having regular adult and child nutrition surveys would be even better.

Policy options that the NZ Government could consider to improve nutrition and reduce inequities in diet-related diseases include:

- Introducing subsidies for healthy food (e.g., provision of vouchers for purchasing discounted fruit and vegetables from farmers markets in low-income communities)^35^;
- Making unhealthy food more expensive (e.g., via food taxes^27^). While such taxes could put more financial burden on low-income households, this can be addressed by using tax revenue to subsidise healthy food and expanding food in school programmes. In addition, tax revenue could be used to subsidise farmers markets in more deprived areas.
- Increasing the regulation around the marketing of unhealthy food (e.g., especially marketing children are exposed to).
- Improving the nutrition-related labelling of foods and mandating warning labels on unhealthy foods (as used successfully in tobacco control, and being legalised in Chile^36^).
- Supporting culturally appropriate Māori led interventions to improve food security and healthy eating in Māori^37,38^ including protecting wild-food resources from waterway and marine pollution.

Future analyses of this data series (when expanded) should also consider the impact of post-2019 food price inflation on food expenditure trends arising from both the Covid-19 pandemic and the war in Ukraine.

## Conclusions

In this study HES data were useful for understanding trends in food expenditure patterns, but limitations remain and further investment in nutrition survey data is recommended. There seems to be slow improvements in diet inequalities by ethnicity, and no evidence of any improvement by income, implying much more must be done to address nutrition to reduce the burden of NCDs and NCD-related inequities in this high-income country.

## Supplementary Information

Supplementary Information.

## Data Availability

Access to the anonymised data used in this study was provided by Statistics NZ under the security and confidentiality provisions of the Statistics Act 1975, https://www.stats.govt.nz/integrated-data/integrated-data-infrastructure/, and so data are not publicly available. Only people authorised by the Statistics Act 1975 are allowed to see data about a particular person, household, business, or organisation, and the results in this paper have been confidentialised to protect these groups from identification and to keep their data safe.
