# Supplementary material for: Using household economic survey data to assess food expenditure patterns and trends in a high-income country with notable health inequities
Source: Sci Rep. 2022 Dec 15;12:21703. doi: 10.1038/s41598-022-26301-z (PMC9753885; doi:10.1038/s41598-022-26301-z)
Supplement: Supplementary file 1 — Supplementary Information. [file 41598_2022_26301_MOESM1_ESM.pdf]

Using Household Economic Survey Data to Assess Food Expenditure Patterns and Trends in a High-Income Country with Notable Health Inequities

Authors: Nhung Nghiem,[1,*] Andrea Teng,[1] Christine Cleghorn,[1] Christina McKerchar,[2] Nick Wilson[1]

[1]Department of Public Health, University of Otago, Wellington, New Zealand

[2]Department of Population Health, University of Otago, Christchurch, New Zealand

[*]nhung.nghiem@otago.ac.nz

Appendix A

Table A1. Characteristics of repeated New Zealand Household Economic Survey samples - Households with any Māori member

Specific metrics	Total sample	2006/07	2009/10	2012/13
Households sample size (n)	1,548	486	555	504
Households included in this study (n)	1,515	471	549	495
Households excluded because they had no food expenditure data (n,%)	24	9	S	12
As above for no income data (zero or negative income)	9	S	S	S
Households members (n)	4,677	1,467	1,731	1,479
Mean age (years)	28.83	28.3	28.37	29.91
Median age (years)	26	27	26	27
Households included any children (n)	756 (48.8%)	234 (48.1%)	279 (50.3%)	243 (48.2%)
All households members aged 65+ years (n)	96	27	30	36
Male (n)	2,205	696	813	696
Female (n)	2,472	768	918	783
Income mean (NZD 2013) per capita	30,381	32,532	27,519	31,524
Income median (NZD 2013) per capita	23,175	23,382	22,842	23,340
Households size (n)	3.09	3.11	3.14	3
Households food expenditure ($)per capita	3,495	3,315	3,399	3,777

Note: S is suppression for sample counts under 6 observations.

Table A2. Characteristics of repeated New Zealand Household Economic Survey samples - Households with all non-Māori members

Specific metrics	Total sample	2006/07	2009/10	2012/13
Households sample size (n)	7,485	2,415	2,568	2,496
Households included in this study (n)	7,392	2,382	2,541	2,472
Households excluded because they had no food expenditure data (n,%)	45	15	12	21
As above for no income data (zero or negative income)	48	21	18	9
Households members (n)	17,973	5,868	6,273	5,835
Mean age (years)	39.54	37.99	39.49	41.15
Median age (years)	40	38	40	43
Households included any children (n)	2,586 (34.5%)	840 (34.8%)	918 (35.7%)	825 (33.1%)
All households members aged 65+ years (n)	1,659	480	546	633
Male (n)	8,688	2,838	3,036	2,814
Female (n)	9,288	3,030	3,240	3,021
Income mean (NZD 2013) per capita	39,174	38,526	39,099	39,879
Income median (NZD 2013) per capita	29,655	29,301	30,078	29,538
Households size(n)	2.43	2.46	2.47	2.36
Households food expenditure ($)per capita	4,230	4,170	4,347	4,170

Appendix B

Table B1. Means of proportion of specific food expenditure out of total annual household income by food group, income-level and ethnicity in three HES waves: 2006/07, 2009/10, and 2013/13.

**Food group**	**Population group**	**Proportion of food specific expenditure out of *total income* (%: mean (se))**		
		**2006/07**	**2009/10**	**2012/13**
**Fruit and vegetables**	Total sample	1.56 (0.04)	1.46 (0.03)	1.53 (0.04)
	Low-income	1.96 (0.07)	1.79 (0.04)	1.88 (0.06)
	High-income	1.01 (0.03)	0.97 (0.03)	1 (0.03)
	Māori	1.12 (0.07)	1.24 (0.06)	1.34 (0.07)
	Non-Māori	1.66 (0.05)	1.52 (0.04)	1.58 (0.04)
**Nuts and seeds**	Total sample	0.1 (0.01)	0.11 (0.01)	0.11 (0.01)
	Low-income	0.12 (0.01)	0.13 (0.01)	0.13 (0.01)
	High-income	0.07 (0)	0.09 (0.01)	0.09 (0.01)
	Māori	0.05 (0.01)	0.06 (0.01)	0.07 (0.01)
	Non-Māori	0.11 (0.01)	0.12 (0.01)	0.12 (0.01)
**Processed meat**	Total sample	0.75 (0.02)	0.77 (0.03)	0.73 (0.02)
	Low-income	0.94 (0.03)	0.96 (0.05)	0.91 (0.03)
	High-income	0.5 (0.02)	0.48 (0.02)	0.46 (0.02)
	Māori	0.85 (0.06)	0.86 (0.05)	0.81 (0.05)
	Non-Māori	0.73 (0.02)	0.74 (0.04)	0.72 (0.02)
**Healthy foods**	Total sample	2.68 (0.07)	2.63 (0.05)	2.62 (0.06)
	Low-income	3.4 (0.1)	3.27 (0.07)	3.26 (0.09)
	High-income	1.71 (0.04)	1.67 (0.04)	1.65 (0.05)

Food group	Population group	Proportion of food specific expenditure out of *total income* (%: mean (se))		
		2006/07	2009/10	2012/13
	Māori	2.2 (0.12)	2.33 (0.12)	2.28 (0.11)
	Non-Māori	2.8 (0.08)	2.7 (0.06)	2.71 (0.07)
**Remaining less healthy foods**	Total sample	11.14 (0.18)	12.32 (0.23)	11.93 (0.23)
	Low-income	13.13 (0.28)	14.78 (0.34)	14.02 (0.36)
	High-income	8.44 (0.16)	8.64 (0.16)	8.75 (0.2)
	Māori	11.27 (0.43)	13.25 (0.46)	13.12 (0.52)
	Non-Māori	11.11 (0.2)	12.08 (0.26)	11.64 (0.27)

Table B2. Relative risks (%) in proportion of household food purchase expenditure for low-income as proportion of high-income and Māori as proportion of non-Māori households, Household Economic Surveys 2006/07, 2009/10, and 2012/13.

Food group	**Population group** (as a proportion of **comparator**)	**Relative risks in proportion of food *group* expenditure out of *total income*** *(%: mean (se))^*		
		2006/07	2009/10	2012/13
**Fruit and vegetables**	Low-income (high-income)	288 (29.25)***	237 (21.36)***	213 (14.25)***
	Māori (non-Māori)	52 (17.74)***	67 (13.77)***	78 (10.41)**
**Nuts and seeds**	Low-income (high-income)	201 (13.63)***	171 (12.02)***	172 (14.03)***
	Māori (non-Māori)	54 (17.92)***	45 (13.71)***	54 (14.7)***
**Processed meat**	Low-income (high-income)	280 (38.31)***	273 (24.87)***	246 (15.37)***
	Māori (non-Māori)	84 (20.72)	92 (14.38)	115 (20.21)
**Healthy foods**	Low-income (high-income)	299 (25.47)***	257 (24.71)***	227 (12.39)***
	Māori (non-Māori)	83 (25.92)	69 (14.61)***	79 (9.35)***
**Remaining less healthy foods**	Low-income (high-income)	230 (27.88)***	232 (20.99)***	195 (16.34)***
	Māori (non-Māori)	76 (15.98)**	84 (12.15)	109 (12.56)

*Note: [a]Values in this table were derived using linear regressions with the survey year and income-level/ethnicity as independent variables. *, **, ***Denote statistical significance at the 10%, 5% and 1% levels, respectively.*

^ Relative risks in proportion of food *group* expenditure out of *total income* must be interpreted with caution as the income variable didn't seem to capture all sources of household income. Food expenditure in some households were greater than their household income, particularly for *non-Māori* households. Other contributions to the odd values (eg, 76% in remaining less healthy food for Māori as proportion of non-Māori) could be household size, and household with children.
